# “‘*We don’t want them to have to live out their lives in the hospital*”: mixed-methods study exploring palliative care needs amongst refugees‘

**DOI:** 10.1177/26323524251317539

**Published:** 2025-02-06

**Authors:** Priya Gupta, Ashlinder Gill, Michael Panza, Olive Wahoush, Humaira Saeed, Jehan Ara Chagani, Christiana Owoo, Christopher Klinger

**Affiliations:** Division of Palliative Care, Department of Family Medicine, McMaster University, Hamilton, ON, Canada; Division of Palliative Care, Department of Family Medicine, McMaster University, 5th Floor David Braley Health Sciences Centre, 100 Main Street West, Hamilton, ON, L8P 1H6, Canada; Division of Palliative Care, Department of Family Medicine, McMaster University, Hamilton, ON, Canada; School of Nursing, Faculty of Health Sciences, McMaster University, Hamilton, ON, Canada; William Osler Health System, Brampton, ON, Canada; Division of Palliative Care, Department of Family Medicine, McMaster University, Hamilton, ON, Canada; Central West Local Health Integration Network, Brampton, ON, Canada; University of Ottawa, Ottawa, ON, Canada; Central West Local Health Integration Network, Brampton, ON, Canada; Division of Palliative Care, Department of Family Medicine, McMaster University, Hamilton, ON, Canada

**Keywords:** access to care, health insurance, palliative care, refugees

## Abstract

**Background:**

The increasing life expectancy and resultant chronic medical comorbidities have resulted in more people requiring palliative care. Unfortunately, palliative care is restricted to marginalized populations, including refugees. In Canada, refugees are only eligible for federal health insurance, which provides basic medical and social coverage until they can obtain provincial health insurance.

**Objectives:**

This study explored limitations in providing palliative care to refugees who had either federal or provincial health insurance in two care settings in Ontario, Canada.

**Design:**

An explanatory sequential mixed-methods approach guided the review of local administrative data and interview data to understand palliative care delivery for refugees.

**Methods:**

Local administrative data from a community health centre and an acute care hospital providing a palliative care approach were collected to review healthcare utilization for refugees with palliative care needs. Interviews from two focus groups with fourteen healthcare providers shared their care experiences in coordinating palliative care for refugees with either federal or provincial health insurance.

**Results:**

Refugee patients with palliative care needs appeared to be accessing acute care services frequently to meet their needs over a 5-year period. Due to a lack of citizenship or permanent residency status, many refugees have access to only federal health insurance. Compared to those with routine provincial coverage, federally insured patients were admitted more frequently. Furthermore, healthcare provider experiences revealed that refugees with only federal insurance coverage had significant barriers to accessing community palliative care support, leading to increased reliance on acute care for quality palliative care.

**Conclusion:**

This study highlights significant gaps in palliative care access for refugees, especially those with federal health insurance. Equitable access is essential in ensuring that patient-centred, quality palliative care is available to all.

## Background

The *Framework on Palliative Care in Canada*—published by Health Canada in 2018—projects that by 2026, the number of deaths in Canada will increase to 330,000 per year and to 425,000 by 2036.^1^ This highlights a pressing need for increasing access to palliative care services; especially for marginalized populations, including Indigenous Peoples, members of the LGBTQ2+ (lesbian, gay, bisexual, transgender, queer/questioning, two-spirited, and others) community, and immigrants and refugees. With increasing attention to inequities in healthcare,^1,2^ the *Framework on Palliative Care in Canada* and subsequent *Action Plan on Palliative Care* identify equitable access to palliative care as one of the guiding principles to improving palliative care in Canada.

### Refugee health

One population facing significant barriers to equitable healthcare access is refugees.^1,3–8^ The 1951 Refugee Convention defines a refugee as “*someone who is unable or unwilling to return to their country of origin owing to a well-founded fear of being persecuted for reasons of race, religion, nationality, membership of a particular social group, or political opinion.*”^9^ Globally, the number of people forcibly displaced unfortunately continues to rise, with close to 1.5% of the entire world population now forcibly displaced, doubling what it was about a decade ago.^10^ Since the 1980s, Canada has welcomed over 1 million refugees,^11^ and consistently is recognized as a world leader in the resettlement of refugees.^12^ Refugees in Canada are divided into two groups: (a) resettled refugees who are selected abroad and become permanent residents once they arrive in Canada and (b) refugee claimants who have fled their country and will receive a decision on their refugee status after they arrive in Canada.^13^ In 2022 alone, 91,740 refugees claimed asylum, with over 26,000 of those in Ontario.^14^

In Canada, healthcare is publicly funded and delivered by provinces/territories for permanent residents. Ontario residents are eligible for provincial health insurance (Ontario Health Insurance Plan). For refugees, they are eligible for federal health insurance (the Interim Federal Health Program) which provides basic medical and social coverage for the first year of arrival or until they are eligible for the provincial health plan.^15^ Resettled refugees often will have concurrent supplemental coverage through federal insurance (for a year) and provincial health insurance as they obtain permanent residency.^16^ For refugee claimants to obtain provincial health insurance, they must wait for a formal hearing to determine their refugee status, a process that can take up to 14 months.^17^

Refugees are largely from low- and middle-income countries with higher rates of chronic health issues.^18^ Research among refugee populations has shown that many refugees have increasing rates of health issues after moving to Canada due to resettlement challenges.^18^ Refugees also experience significant healthcare barriers, including language and cultural barriers, unfamiliarity with the healthcare system, and increased financial burden.^3,7,8^ There are limited studies assessing palliative care delivery in refugee populations. An Ontario study demonstrated that recent immigrants were 15% more likely to use aggressive end-of-life care and 5% less likely to receive supportive care.^4^ A case report from Toronto, Ontario revealed insurmountable barriers in providing palliative care to a refugee patient under the constraints of the federal health plan, resulting in an eleven-month hospital admission.^8^ Countries with similar publicly funded healthcare systems such as Australia revealed access to palliative care being significantly challenged due to a lack of eligible health insurance. This could lead to an overreliance on care provided by volunteers and/or delayed referrals to palliative services.^19^ Despite the challenges, international studies highlighted the importance of integrating palliative care in the care of refugees as a palliative approach to care led to an increased sense of belonging to society, assisted in recovery from trauma and alienation, and bridged the cultural gap between population and host communities.^5,20^

Thus, the goal of this study was to conduct a preliminary investigation that explores barriers to and facilitators in providing outpatient palliative care for refugees in the Greater Toronto and Hamilton area in Ontario (Canada) when having either provincial or federal health insurance.

## Methods

### Study design

An explanatory sequential mixed method design^21,22^ was utilized by first reviewing administrative data of refugee patients who had a palliative diagnosis and were seen by the community primary care team or acute care-based palliative care team. Quantitative data described healthcare utilization and demographic information to characterize this population and with the purpose to determine if patterns of healthcare use across insurance status exist. To better understand how insurance status impacts access to palliative care, qualitative interviews provided additional explanation of barriers and facilitators to accessing care, and underlying mechanisms which may hinder or support access. Collecting both quantitative and qualitative data facilitated a more fulsome understanding of access to care from healthcare provider perspective. All data demonstrated cross-validation of palliative care needs and information that may not have been available from one method of data collection. The reporting of this study conforms to the MMAT mixed-methods statement (Supplement A).^23^ Integration of all data was reviewed during analysis, where identified topics specific to insurance status and access to care aided the explanation of barriers and facilitators to accessing palliative care. To ensure a sequential exploratory mixed-methods design was applied during data analysis and integration, *contiguous integration of* both qualitative and qualitative data is presented in separate sections to first understand refugee access to acute care, then later explore *why* this pattern of care may exist.

### Setting

The study was based at two sites from two different municipalities: a community health centre and an acute care hospital. A community health centre is a unique model of providing healthcare to underserved populations, including refugees, by emphasizing a multidisciplinary approach to community health, and equitable access to primary and social care.^24^ The acute care hospital is a full-service community hospital providing a wide range of inpatient and outpatient services, including an interdisciplinary palliative care consultant team. Both sites were ideal to explore access to palliative care for refugee patients due to their experience working with refugee claimants in both outpatient and inpatient medical settings. Furthermore, both care teams are situated in more urban geographic regions that have greater refugee settlements.

### Data collection

Routinely collected administrative data was obtained from Integrated Decision Support and local electronic medical record databases at both sites to review and analyze indicators of palliative care access, including the emergency department visits (National Ambulatory Care Reporting System), hospitalizations (Discharge Abstract Database), and referrals to home and community care services (Client Health and Related Information System) from January 1, 2017 to January 1, 2023.^25,26^

Both sites included active, inactive, transferred, or deceased patients who were 18 years of age and older, had either Interim Federal Health Plan (federal) or provincial healthcare insurance and were diagnosed with one or more chronic diseases from a list of 26 International Classification of Diseases codes or International Classification of Primary Care chronic disease codes (Supplement B).

Focus groups of multidisciplinary healthcare providers were conducted to better understand care organization and the specific needs of refugees with life-limiting illnesses. Focus groups were heterogeneous to ensure representation across healthcare professionals and conducted by one facilitator using a semi-structured interview guide, and one observer. Focus groups were audio-recorded and transcribed verbatim. Individual interviews were conducted with healthcare providers who functioned more independently in their practice, and not a part of a team-based practice. Informed consent was conducted via written form and verbal agreement prior to recording. To ensure participant anonymity, transcripts were de-identified by study team members. Purposeful sampling was employed to ensure all healthcare providers who coordinated palliative care across these healthcare teams were interviewed about their care experiences.^27^ Once sampling was complete, inductive thematic saturation of experiences related to care coordination from the perspective of outpatient healthcare providers was achieved, as all providers were interviewed, and no new topics emerged with each preceding interview.^28^

### Data analysis

For quantitative data, summary descriptive statistics were computed using SPSS Version 28; mean and standard deviations were calculated for the continuous variables. Significant differences were not tested due to sample size.

For qualitative data analysis, a qualitative descriptive approach was used to describe participants’ experiences.^29^ Inductive content analysis^30^ helped identify the context of delivering palliative care to refugees. Data was coded inductively by team members and described with labels to group similar feedback together. Inductive coding analysis was used in the first stage to identify categories addressed by participants. These categories were summarized early in the analysis process and agreed upon by team members by consensus discussion.^31,32^ When all data had been coded, members of the analysis team met and applied content analysis to group categories together, based on how experiences were similar (or different) across participants. From the complete codebook (Supplement C), themes specific to *funding, systemic/structural factors*, and *care coordination* were reviewed in greater detail as secondary coding, for the purpose of this investigation. Data was managed and coded in NVivo Version 14 and summarized with all team members by consensus.^30^ To ensure a rigorous approach, additional techniques were utilized, including bracketing, reflexivity, and audit trails to draw upon analytic and clinical expertise from multidisciplinary team members and ensure bias was recognized and addressed.^33^

## Results

### Administrative data

Administrative data from both sites identified 505 refugees who received care between January 1, 2017, and January 1, 2023. Of these, 37 refugee patients from the acute care site and 34 refugee patients at the community site had a palliative care need (14% of the total sample of refugee patients; Table 1).

**Table 1.** Characteristics of refugee patients who sought palliative care.

Sample characteristic	Cases		
	Community health centre (*N* = 34)	Acute care (*N* = 37)	Total (*N* = 71)
Demographics			
Male	21 (62%)	21 (57%)	42 (59%)
Age, years (mean (SD))	45 (15.01)	60 (15.2)	53 (±16.8)
Age, years			
20–50	23 (68%)	11 (30%)	34 (48%)
51–70	9 (26%)	16 (43%)	25 (35%)
71+	2 (6%)	10 (27%)	12 (17%)
Insurance status			
Only IFHP	1 (3%)	29 (78%)	30 (42%)
Only OHIP	7 (21%)	8 (22%)	15 (21%)
Both IFHP and OHIP	26 (77%)	—	26 (37%)
Disease			
Liver disease	19 (56%)	5 (14%)	24 (34%)
Renal disease	5 (15%)	10 (27%)	15 (21%)
Cancer	2 (6%)	26 (70%)	28 (39%)
Stroke	1 (3%)	4 (11%)	5 (7%)
Dementia	3 (9%)	5 (14%)	8 (11%)
CHF	3 (9%)	8 (22%)	11 (15%)
Other*	5 (15%)	3 (8%)	8 (11%)
Chronic conditions			
1	31 (91%)	15 (44%)	46 (65%)
2	2 (6%)	12 (35%)	14 (24%)
3	1 (3%)	6 (18%)	7 (15%)
4	0	1 (3%)	1 (1%)

* IPF and ILD, IHD, COPD, or Lung Disease.

CHF, congestive heart failure; COPD, chronic obstructive pulmonary disease; IFHP, Interim Federal Health Program; IHD, ischaemic heart disease; IPF, idiopathic pulmonary fibrosis; ILD, interstitial lung disease; SD, standard deviation; OHIP, Ontario Health Insurance Plan.

Patients were on average 53 years of age (±16.8), slightly younger than the average population that accesses palliative care in Ontario.^34^ Refugees accessing the community health centre often had both federal and provincial health coverage (77%), whereas refugees accessing the acute care site only had federal coverage (78%). At the community health centre, refugees mostly presented with liver disease (56%); the acute care setting had more cancer diagnoses (70%). This is likely due to community patients being slightly younger, as more acute care patients also had greater multiple chronic conditions.

Healthcare utilization was also assessed (Table 2).

**Table 2.** Healthcare utilization.

Sample characteristic	Cases		
	Community health centre (*N* = 34)	Acute care (*N* = 37)	Total (*N* = 71)
	(*n* = 7)	(*n* = 32)	(*n* = 39)
Hospitalizations			
Number of visits (range)	22 (1–11)	73 (1–9)	95
Mean (SD)	3.14 (3.27)	2.28 (1.96)	2.43 (2.23)
		(*n* = 32)	
Length of Hospital Stay (Days)			
Mean (SD)	—	45.1 (52.25)	—
Minimum	—	4	—
Maximum	—	273	—
	(*n* = 19)	(*n* = 25)	(*n* = 44)
Emergency Department Visits			
Number of visits (range)	186 (1–48)	124 (1–53)	310
Mean (SD)	9.79 (12.53)	4.96 (10.23)	7.05 (11.27)

A total of 39 of 71 patients required hospitalizations: 32 patients from acute care compared to 7 patients from the community. These patients had an average of 3.14 (±3.27) hospitalizations over a 5-year period versus acute care patients having an average of 2.28 (±1.96) hospitalizations. In addition, a total of 44/71 patients visited the emergency department over this time. Community patients had 19 patients access the emergency department with an average of 9.79 (±12.53) visits over the last 5 years. Acute care patients had an average of 4.96 (±10.23) visits over the same period.

Healthcare utilization was also compared by insurance status (Table 3).

**Table 3.** Insurance status and healthcare utilization.

Healthcare utilization	Cases				
	*Community Health Centre Setting* (*N* = 34)			*Acute Care Setting* (*N* = 37)	
	Only IFHP (*n* = 1)	Only OHIP (*n* = 7)	Both (*n* = 26)	Only IFHP (*n* = 29)	Only OHIP (*n* = 8)
	*n* (%)	*n* (%)	*n* (%)	*n* (%)	*n* (%)
Hospitalizations (1 or more)	0 (0)	1 (14.3)	6 (23.1)	25 (86.2)	7 (87.5)
Emergency Department Visits (1 or more)	0 (0)	4 (57.1)	15 (57.7)	20 (69)	5 (62.5)
Emergency Department Visit Range (Days)					
1–5	0 (0)	3 (75)	7 (46.7)	17 (85)	5 (100)
6–20	0 (0)	1 (25)	6 (40)	2 (10)	0 (0)
21+	0 (0)	0 (0)	2 (13.3)	1 (10)	0 (0)
Hospital Length of Stay					
1 week or less				5 (20)	1 (14.3)
2 weeks				3 (12)	2 (28.6)
3 weeks				1 (4)	1 (14.3)
4 weeks				3 (12)	0 (0)
Over 4 weeks				13 (52)	3 (42.9)

Of community patients who had both insurance coverage, 6 patients (23%) required hospitalizations and 15 patients (57.7%) visited the emergency department. Some patients (8/15) reported more than 6 trips to the emergency department over the last 5 years. Acute care patients with only federal insurance (29/37 patients) and only provincial coverage (8/37 patients) were also compared. While the majority of patients on both insurance coverages visited the emergency department 1–5 times, those with federal insurance had 3 patients visiting the emergency department more than 6 times. For those with only federal health insurance, the majority of patients (17/25) had a hospital length of stay greater than 2 weeks, with 13 patients of those patients (52%) having a hospital stay greater than 4 weeks. In contrast, 3/7 (42.8%) provincially-funded patients had a stay greater than 4 weeks.

### Qualitative data

**Figure 1.**
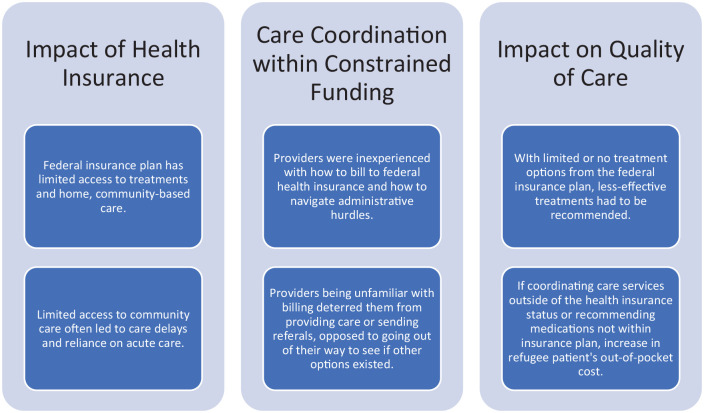
Identified themes specific to refugee health insurance status and access to palliative care.

Across both focus groups from each site, a total of fourteen healthcare providers shared their experiences specific to managing the palliative care needs of refugees. Eight healthcare providers participated from the acute care consult team (three physicians, one nurse, two nurse practitioners, and two social workers). Six providers participated from the community site (one physician, one social worker, one nurse practitioner, one registered practical nurse, one pharmacist, one client advocate). To better understand the experiences of healthcare providers managing the palliative care needs of refugees, the following themes from the complete codebook (Supplement C) are discussed in greater detail; impact *of health insurance, care coordination within constrained funding*, and *impact on quality of care* (Figure 1).

### Impact of health insurance

Participants highlighted that a refugee’s health insurance status was often a barrier to accessing healthcare. Most commented that having only federal health insurance limited access to specific treatments and care, resulting in lengthy hospital stays.

This individual did not—he was without status at all, and so . . . he ended up in the hospital for an extremely lengthy admission accruing significant costs for his unfunded care here . . . And he had been in the hospital for many months, had been admitted and re-admitted several times before his long length of stay. And one of the barriers to him being able to be in the community was because he had such fragile health and such specific needs . . . that without support and intervention he wouldn’t function well in the community. And he couldn’t access what he needed. [Acute Care Focus Group—Social Worker]

In Ontario, provincial homecare services require an active provincial health insurance number to access services, including most hospice care. Thus, those with federal insurance are often precluded from accessing home care through traditional provincial organizations. Acute care providers specifically commented that while the federal insurance has some homecare coverage for personal support workers, nursing, and equipment, finding a provider for these supports was challenging as providers must be registered to bill through a specific insurance company to process these claims.

I have actually two people on Interim Federal Health Cards [federal health insurance]—two refugees . . . more recently I had one in [city] and this lady died, it was very unfortunate . . . I was a nurse practitioner in an out-patient program with an oncology team and I got a call just before the weekend saying, “Things were pretty poor.” She ended up in the hospital and she died there. But when I phoned the care coordinator because somebody had brought her on service for homecare she says, “No, no, no, she’s a—you know—that refugee status, she’s on an Interim Federal Health Card, we really don’t have anything to do with her.” This is what the care coordinator said to me in [city], and I said, “I find that really hard to believe. Let’s work together to see what we can do.” I said, “Please give me the number for the agency that’s responsible for her services.” Which she did and I won’t disclose the agency, but I did call them and this person’s telling me, “We have to assess her eligibility for service.” I said, “You’ve had weeks to do that. . . [Community Health Centre—Nurse Practitioner]

This additional administrative step often resulted in care delays and refugees not receiving support at home.

Community providers commented on limited funding for certain medications and treatments, further burdening out-of-pocket costs for refugees. While federal health insurance covers the cost of medications under the Ontario Drug Formulary, federal insurance often requires pre-authorization before funding a specific medication. One participant also commented on the challenges of drug coverage for refugees who transition from federal to provincial health insurance and subsequently lose drug coverage.

And then the drug coverage is even a step above that, because quite often they’re in a low income category, but they’re in a working poor—I guess you could say, and they need to apply for Trillium [provincial drug plan] ‘cause they’re not on Ontario disability and things. So, then they have to go through that process over and above their OHIP [provincial health plan] process. [Community Health Centre Focus Group—Pharmacist]

Such circumstances left refugees and their families unable to access first-line treatments, often resorting to second- or third-line therapies that were financially more viable. Once again, this additional administrative hurdle resulted in suboptimal care.

### Care coordination within constrained funding

Due to poor access to community care and compromised treatment plans, healthcare providers from both sites experienced challenges with coordinating care. As the federal and provincial insurance systems are different, many providers are unaware of how to register and bill their services through the federal program and may be inclined to not provide services. Furthermore, with patients being ineligible for specific treatments, diagnostic testing, or home care, the organization or patient often absorbed these additional out-of-pocket costs. Acute care participants highlighted the need of having a “navigator” on the patient’s care team as a potential facilitator in addressing these administrative hurdles. This role was repeatedly mentioned by providers who worked within the acute care setting, especially when discharging patients to the community.

Instead of palliative [patients] going home on OHIP [provincial health plan] [who] are provided with [their] care-coordinators name and phone number, the list of their different service providers . . .—like what’s the nurses phone number, what’s the Occupational therapists phone number? Equipment is delivered before they—you know . . . are discharged. Like so, they’re given details and phone numbers that they can call, and it feels like the refugee folks are more left . . . on their own [Acute Care Focus Group—Hospital Care Coordinator]

The community site described a “genie” fund to help cover some out-of-pocket costs and assist in referring refugee patients to community organizations that provide interest-free loans for managing complex health and social needs.

### Impact on quality of care

Participants consistently commented on several encounters where recommendations on care plans and treatments had to be modified due to lack of health coverage. One participant described an extreme example where the patient was advised to return to their home country for cancer treatment.

There was one instance a while back where one of our doctors actually had to have a client return to her country because she had a very advanced cancer, and she had no medical coverage. So, his best recommendation was for her to return home where she had full coverage to get treatment. That’s a sad situation, but the client understood that she couldn’t stay in Canada and be treated. So, she went back home. [Community Health Centre Focus Group—Registered Practical Nurse]

Many healthcare providers expressed distress around the health system consistently failing refugees with palliative care needs.

We don’t want them to have to live out their lives in the hospital. But it’s just when we see these things fall—falling apart and the-the-the stress and the turmoil that families end up in when things are not working out because there wasn’t that foresight or there—the people like—case management didn’t know about these things and how to support them. That—that traumatizes people, too. [Acute Care Focus Group—Physician]

While providers aimed to provide holistic and comprehensive care, they felt constrained and restricted due to administrative barriers, leading to decreased quality of care. Healthcare providers expressed feeling helpless, knowing the system was leading to further traumatization of an already vulnerable group.

## Discussion

### Main findings

This study is the first to look at how refugees in Canada access palliative care. Integrating both administrative data and healthcare provider interviews demonstrated an overreliance on acute care services to support palliative care needs, due to limited coverage of medications and community-based care with the federal health insurance for refugee patients. Both data sources help to expand insights and provide a complementary understanding of accessing care. In this sample of refugee patients with a palliative care need, those with federal health insurance had relatively higher numbers of emergency department visits, hospitalizations, and increased length of stay than those with provincial insurance. This was further highlighted in the qualitative portion of our study as focus groups identified and partially explained possible reasons including poor access to home and community care. Increased acute care usage due to a possible lack of healthcare funding has been identified in similar studies looking at access to palliative care for newcomers and undocumented immigrants in Canada.^3,6^

Furthermore, healthcare provider experiences provided additional explanations for the perceived increasing reliance on acute care to meet palliative care needs. In Ontario, community palliative care support, including nursing, personal support workers, equipment, and care coordination, is primarily organized through provincial institutions. Patients who do not have an active provincial health insurance number are unable to access this central service for palliative care support. While federal insurance covers about 140 hours per month of personal support workers, community nursing visits, and home-based equipment (such as commodes and hospital beds),^15^ many homecare organizations do not provide care for refugees with federal insurance due to issues and delays with compensation through the federal health plan. This is further complicated as many Ontario hospices do not accept patients without provincial health insurance coverage. As reflected in our findings, this leads to unreliable and fragmented access to palliative care support in the community.

### What this study adds?

This study highlights the need for ongoing education about federal insurance coverage to providers who may not routinely work with refugee patients. Many providers expressed helplessness when caring for refugees as they felt they were forced to deliver suboptimal palliative care. Education is especially important for providers who work in hospitals and those involved in discharge planning for refugees. Providers highlighted access to a care coordinator, akin to coordinators offered through provincially funded homecare, to help patients navigate a complex system with federal insurance, or provide additional resources to help pay for out-of-pocket costs. Understanding the local landscape with respect to dedicated community providers who see patients who are federally insured can help discharge planners coordinate care and ensure a safer discharge home. Previous literature suggests that a strong care coordinator role can reduce acute care utilization, lower patient and family anxiety, and help to improve overall quality of life.^35,36^ A designated coordinator for refugees can help reduce the need for hospitalizations to meet care needs for those approaching end-of-life.

Despite federal healthcare providing coverage for much of the basic homecare support for patients with palliative needs, our study indicates that these supports are not easily accessible. This problem is unfortunately not unique to Canada, as other countries with publicly funded homecare including Australia^19^ and Germany^37^ face similar challenges. Patients with palliative care needs often change quickly and have increasing needs as they approach end-of-life. Delays in reimbursement and care organization often lead to disruption in service, equipment, and medication delivery, thus increasing the risk of uncontrolled symptoms and caregiver distress. Access to expedited administrative processes for refugees with palliative care needs could help mitigate delays and ensure consistent and adequate community support. Additional research is warranted to review palliative care access for refugees in larger administrative datasets to compare both insurance types and access to care. Federal health claims data could help highlight current homecare services for the population on a broader scale and inform system optimization—including easier provider access and remuneration.

### Strengths and limitations of the study

This is the first study to review the palliative care needs of refugees in Canada. Although the sample size and data were limited to two select areas, it is the first to review healthcare utilization and challenges specific to insurance and care organizations. A mixed-methods approach was able to contextualize why refugees may rely on acute care services when managing advanced illness, and the limitations of coordinating home and community-based supports.

It was difficult to ascertain how many patients had access to community support, as this information was not readily available through local datasets. Our comparison of acute care usage only included refugee patients with either insurance type but did not include patients who were not refugees and had provincial coverage. A final limitation is the lack of refugees and/or family members included in the qualitative portion of the study. Unfortunately, recruitment in this population group proved to be quite challenging, due to issues with scheduling, technology, and translation. It is also recognized that refugees are a heterogeneous group and come from diverse communities, and while the findings in this study are generalized, they may not be relevant to all cases.

## Conclusion

Findings suggest that refugees, especially those with only federal health coverage, often access acute care services to meet their palliative care needs. Issues with funding, coordinating community providers, and administrative burdens have created significant barriers for refugees to access palliative care. Proposed solutions include federal coverage education for providers, establishing a care coordinator, and advocating for more streamlined processes for refugees with palliative care needs. Future qualitative work should also include the refugee patient/family perspective to further understand the patient experience.

## Supplemental Material

sj-docx-1-pcr-10.1177_26323524251317539 – Supplemental material for “‘We don’t want them to have to live out their lives in the hospital”: mixed-methods study exploring palliative care needs amongst refugees‘ Supplemental material, sj-docx-1-pcr-10.1177_26323524251317539 for “‘We don’t want them to have to live out their lives in the hospital”: mixed-methods study exploring palliative care needs amongst refugees‘ by Priya Gupta, Ashlinder Gill, Michael Panza, Olive Wahoush, Humaira Saeed, Jehan Ara Chagani, Christiana Owoo and Christopher Klinger in Palliative Care and Social Practice

## Declarations

**Ethics approval and consent to participate:** The study was approved by the Hamilton Integrated Research Ethics Board (HiREB #14182) and the William Osler Research Ethics Board (#22-0042). All study participants provided informed consent.

**Consent for publication:** Informed consent included consent for the utilization of anonymized quotes for publication. Preliminary results were presented, for example, at the 2023 Advanced Learning in Palliative Care Conference in Calgary, Alberta in May, the 2023 Hospice Palliative Care Ontario Conference in Richmond Hill, Ontario in June, the 2023 North American Refugee Health Conference in Calgary, Alberta in August, and the 2023 Canadian Hospice Palliative Care Conference in Ottawa, Ontario in October.

**Availability of data and materials:** The data that support the findings of this study are available on request from the corresponding author, AG. The data are not publicly available due to the anonymity of participating facilities and healthcare providers. Therefore to ensure there is no privacy breach, de-identified data can be provided upon request.
