# Supplementary material for: “‘We don’t want them to have to live out their lives in the hospital”: mixed-methods study exploring palliative care needs amongst refugees‘
Source: Palliat Care Soc Pract. 2025 Feb 6;19:26323524251317539. doi: 10.1177/26323524251317539 (PMC11803610; doi:10.1177/26323524251317539)
Supplement: sj-docx-1-pcr-10.1177_26323524251317539 – Supplemental material for “‘We don’t want them to have to live out their lives in the hospital”: mixed-methods study exploring palliative care needs amongst refugees‘ [file sj-docx-1-pcr-10.1177_26323524251317539.docx]

Supplement A: MMAT Mixed Methods Appraisal Tool

| Category of study design | Quality criteria |  |
| --- | --- | --- |
| 1. Qualitative Descriptive | 1.1. Is the qualitative approach appropriate to answer the research question? | Research question is to explore barriers and facilitators to accessing palliative care, when refugees have specific federal or provincial health plan (page 4). |
|  | 1.2. Are the qualitative data collection methods adequate to address the research question? | To get a deeper understanding of barriers and facilitators to assessing care, in-depth interviews and focus groups were conducted with healthcare providers who coordinate care and shared their experiences of health system navigation for refugee patients. Focus groups were conducted with healthcare providers who work directly together and individual interviews were conducted with providers who coordinate care more independently in the community. (Page 5/6) |
|  | 1.3. Are the findings adequately derived from the data? | Inductive coding ensured identified themes and topics from participant experiences were summarized in the codebook. (page 6) |
|  | 1.4. Is the interpretation of results sufficiently substantiated by data? | Exemplary quotes have been provided for each theme that are rich in narrative description. (pages 10 – 13) |
|  | 1.5. Is there coherence between qualitative data sources, collection, analysis and interpretation? | Data source is healthcare provider interviews that include experiences of coordinating palliative care for refugee patients. Analysis reflects descriptive coding to describe barriers to and facilitators in accessing care, and the impact of having a specific type of health insurance. |
| 4. Quantitative Descriptive | 4.1. Is the sampling strategy relevant to address the research question? | Sampling strategy was to review refugee patients with life-limiting diagnosis who received care from two health care teams to understand their needs and patterns of healthcare access. (Page 3) |
|  | 4.2. Is the sample representative of the target population? | The sample of patients reviewed from administrative data included specific inclusion and exclusion criteria, using disease codes for life-limiting diagnosis and refugee status. (page 5) |
|  | 4.3. Are the measurements appropriate? | International Classification of disease codes were references for disease codes commonly used to characterize patient population. Refugee status is reflected by federal insurance status and listed within demographic profiles within healthcare administrative records. (page 5) |
|  | 4.4. Is the risk of nonresponse bias low? | Given the data is retrospective review of administrative data, response rate was a non-issue. |
|  | 4.5. Is the statistical analysis appropriate to answer the research question? | Descriptive data analysis was completed to describe the patient population. A low sample size hindered inferential statistics for additional comparison. |

Hong, Q. N., Gonzalez-Reyes, A., & Pluye, P. (2018). Improving the usefulness of a tool for appraising the quality of qualitative, quantitative and mixed methods studies, the Mixed Methods Appraisal Tool (MMAT). *Journal of Evaluation in Clinical Practice*, 24(3), 459-467.

Supplement B: Disease Classification

| Code | Code Type | Condition |
| --- | --- | --- |
| B90 | ICPC | HIV/AIDS |
| C00-C97 | ICD-10 | Cancer |
| K74 | ICPC | IHD |
| K75 | ICPC | IHD |
| K76 | ICPC | IHD |
| K77 | ICPC | CHF |
| K78 | ICPC | CHF |
| K79 | ICPC | CHF |
| K80 | ICPC | CHF |
| K81 | ICPC | CHF |
| K82 | ICPC | CHF |
| K83 | ICPC | CHF |
| K84 | ICPC | CHF |
| K90 | ICPC | Stroke |
| K91 | ICPC | Stroke |
| R95 | ICPC | COPD |
| J84.1, J84.9 | ICD-10 | IPF, ILD, Other Respiratory*  Diseases |
| D97 | ICPC | Liver Disease |
| U99 | ICPC | Renal Disease |
| P70 | ICPC | Dementia |
| N87 | ICPC | Parkinson's |
| N86 | ICPC | MS |
| G12.2 | ICD-10 | ALS, Other Neurological Diseases** |

**Exclude Encode 6141 pneumonitis*

****exclude Encode 4742 pseudobulbar palsy*

Supplement C: Complete Codebook

| **Code Name** | **Code Description** |
| --- | --- |
| **Systemic/Structural** | |
| Acute Care | Hospital-based coverage during pandemic, IFHP covers acute care, refugee claimants have to pay out-of-pocket since no IFHP/OHIP develop payment plan; intensification of care at EOL |
| Funding | When a client/patient can apply for different insurance plans, implications of the funding, dictates level of access, blanket “pandemic” coverage for acute care-based care |
| Systemic/structural factors | IFHP provides lifetime 40 hours of nursing; no PSW/OT or IV meds; refugees cannot access community hospices; equipment, services; getting the care they need, when they need it. |
| **Healthcare Provider Refugees and Palliative Care** | |
| Care coordination | Providers have various experiences and responsibilities, depending on their professional background and if they can provide additional language support to a specific group of refugees CHC-specific resources |
| Patient intake | How refugee clients are “assigned” or referred to providers within CHC |
| Provider roles  Good Samaritans | Providers have various experiences and responsibilities, depending on their professional background and if they can provide additional language support to a specific group of refugees |
| Palliative approach to care | Awareness, understanding, knowledge, training, professional development for clinicians |
| **Refugee Patients/Family Caregivers and Access to Care** | |
| Social determinants of health | Social support, Income/Financial burden, challenges related to housing, language barriers, past trauma and impact on mental health during settlement. |
| Psychosocial support | Family caregivers burdened by managing health and social care needs, along with pressures of being newly settled in a new country, language and culture. |
| Perceptions, cultural understanding | Specific to healthcare, self-care, preventative care, or scheduled appointments. Understanding of advanced disease and disclosing health needs, health literacy. Fear to disclose health needs, may jeopardize their application/paperwork. Poor understanding of palliative/end-of-life care, not likely to choose hospice care. Different experiences in home country compared to Western model of care. Preference for nontraditional medicines |
| System navigation | Lack of familiarity with healthcare system; Knowing where to go for their needs/concerns, ability to interact with health system. Facing medical jargon. |
